# Epidemiological and Clinical Characteristics of Intussusception in Children Aged Under 2 Years: Comparison of the Southern and Western States in India, 2019–2022

**DOI:** 10.1007/s12098-025-05784-4

**Published:** 2025-11-10

**Authors:** Varsha Sudhir Chaudhary, Namrata Kharat, Anupama Machathi, Ragavi Lingam, Poovarasan Kannan, Ayyapan Vellathur Ramasamy, Prasanna Samuel Premkumar, Venkata Raghava Mohan

**Affiliations:** 1 Department of Community Medicine, Christian Medical College, Vellore, Tamil Nadu, India; 2 Department of Biostatistics, Christian Medical College, Vellore, Tamil Nadu, India

**Keywords:** Intussusception, Pediatric intestinal obstruction, Kerala, Karnataka, Maharashtra, Gujarat

## Abstract

**Objectives:**

To compare the epidemiological and clinical characteristics, management, and outcome of intussusception in children in the southern and western states in India.

**Methods:**

This multicenter observational study, conducted in 21 hospitals between August 2019 and December 2022, compared the characteristics of intussusception among children younger than 2 y hospitalized with intussusception in the southern (Kerala and Karnataka) and western (Maharashtra and Gujarat) states in India. Children were diagnosed with intussusception according to level 1 Brighton Collaboration criteria.

**Results:**

Of the 1,319 children enrolled, 860 (65.2%) and 459 (34.8%) were from the southern and western states, respectively. Clinical manifestations were similar in both regions; however, children from the southern states exhibited more frequent incessant crying (32.2% vs. 1.3%; *p* < 0.001). The predominance of ileo-colic presentation was significantly more marked in the southern states vs. the western states (91.7*% vs.* 80.6%; *p* < 0.001). Significantly more cases were resolved by radiological reduction in the southern states (89.2% vs. 56.9%; *p* < 0.001), whereas surgical intervention was more common in the western states. The case fatality rate was 0.3%. All four deaths occurred in the western states (*p* = 0.022).

**Conclusions:**

The poorer outcome in the western states might be attributable to longer delays in seeking treatment, and disparities in healthcare access. These regional divergences highlight the differences in healthcare infrastructure, and socioeconomic inequities between regions. Managing these differences through health education, improving access to diagnostic tools, and timely intervention could reduce the burden of intussusception in India.

## Introduction

Intussusception is a common critical pediatric gastrointestinal emergency, characterized by the telescoping of one segment of the intestine into an adjacent segment, leading to bowel obstruction and ischemia, with the potential for life-threatening complications such as perforation [1, 2]. The incidence is highest in children aged under 2 y, with the peak incidence in infants aged 6 to 12 mo [3, 4]. The condition has a non-specific clinical presentation, with signs such as abdominal tenderness, vomiting, and red currant jelly stools, which can complicate early diagnosis, thus delaying intervention [1, 5]. Intussusception is clinically important not only because it can be life-threatening but also because of its association with rotavirus vaccination [6–8].

A literature review of 82 studies by Jiang et al. [5], reported diverse epidemiology, clinical presentations, treatment methods, and outcomes in different geographical regions. Geographical variation in the incidence and management of intussusception suggest that factors like environmental, genetic, and healthcare infrastructure may influence both the incidence and outcomes of intussusception in India [3, 9]. Studies in southern India by Jehangir et al. [9], and northern India by Bahl et al. [10] have reported an incidence of intussusception ranging from 17.7 to 254 cases per 100,000 child-years. Other studies in the Indian population have shown regional variability in the healthcare infrastructure and modalities used to treat intussusception. In addition, the southern region has a higher adult literacy rate and better healthcare access, compared with the northern and western regions [11], which have multiple healthcare challenges, affecting both early diagnosis and management of pediatric emergencies such as intussusception [8, 9]. The authors hypothesized that these regional differences could lead to regional variations in the diagnosis and management of intussusception, thereby affecting its overall outcome.

Therefore, they compared the epidemiological and clinical characteristics, management, and outcome of intussusception in children younger than 2 y in the southern and western states in India. The findings could contribute to a deeper understanding of regional disparities in pediatric healthcare system and guide strategies to improve the prevention, early diagnosis, and management of intussusception.

## Material and Methods

This analysis was part of a large multicenter observational study to assess the safety of ROTASIIL after its introduction in the Universal Immunization Program in the four Indian states of Kerala and Karnataka in southern India and Maharashtra and Gujarat in western India. It was conducted at 21 tertiary care hospitals with in-patient facilities for diagnosing and treating confirmed cases of intussusception between August 2019 to December 2022.

All children younger than 2 y, who fulfilled the case definition of intussusception according to the level 1 Brighton Collaboration criteria [12] and who met the eligibility criteria [13] were enrolled in the study after obtaining written informed consent from the child’s parent or guardian. Each enrolled child was assigned a unique enrollment number, and the copies of ultrasound reports, discharge summaries, ultrasound images (pre- and post-treatment), and immunization history were collected.

Statistical analysis was performed using Stata version 16.1 (StataCorp LLC, College Station, TX, USA) [14]. The data were summarized as frequencies and percentages or as medians and interquartile ranges. The statistical significance of differences between groups was assessed using the rank-sum test, chi-square test, or Fisher’s exact test, as appropriate. Two-tailed *p* values less than 0.05 were considered statistically significant.

All necessary permissions, including ethical approval, were obtained from the Institutional Ethical Committee of each participating site after approval by the Institutional Review Board of the national coordinating center.

## Results

**Fig. 1:**
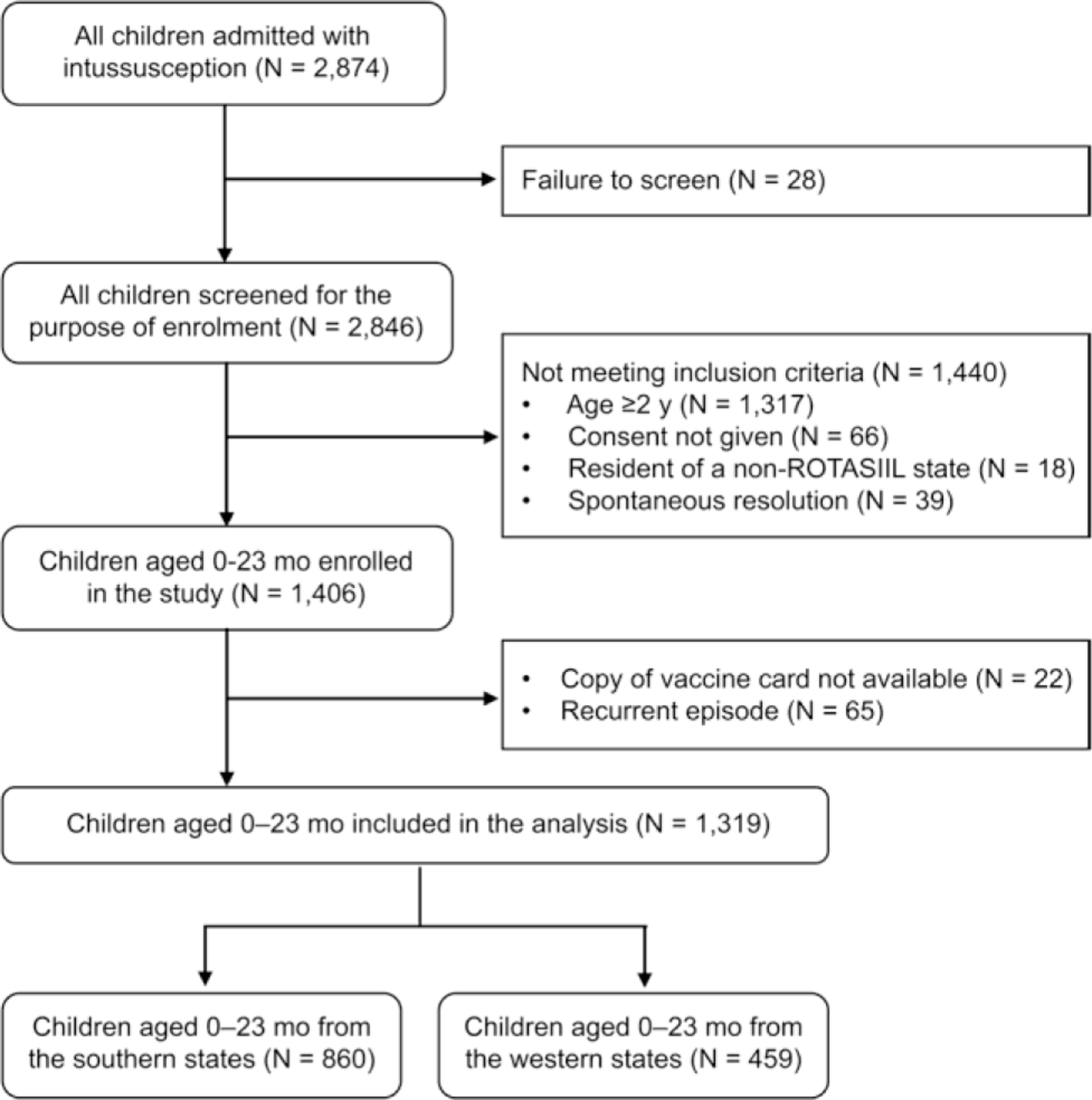
Flow chart of the enrolment of children aged younger than 2 y with intussusception in the southern (Kerala and Karnataka) and western (Maharashtra and Gujarat) states in India

A total of 1,319 (45.9%) children younger than 2 y were enrolled from among 2,874 children admitted with intussusception during the study period, of whom 860 (65.2%) were from the southern states and 459 (34.8%) were from the western states (Fig. 1). The median age of the children from the western states was lower than that of the children from the southern states (8 vs. 9 mo, *p* = 0.025). The proportion of affected infants was higher in the western states than in the southern states (69.7% vs. 60.5%; *p* = 0.001). The sex distribution was comparable in the southern and western states, with a predominance of boys in both regions (63.4% vs. 65.4%; *p* = 0.474). The proportion of children treated in private hospitals was similar in the southern and western states (42.9% vs. 39.0%; *p* = 0.170). The wealth distribution varied significantly by region, with a greater proportion of children belonging to the lower wealth tertile in the western states than in the southern states (47.5% vs. 32.6%; *p* < 0.001). The proportion of children who were admitted directly to the study hospital was significantly higher in the western states than in the southern states (68.6% vs. 62.0%; *p* = 0.016) (Table 1).

Vomiting, abdominal tenderness, blood in stools, diarrhea, fever, and constipation were significantly more frequent in children from the western states (all *p* < 0.001), whereas incessant crying was significantly more common in children from the southern states (32.2% vs. 1.3%; *p* < 0.001). The predominance of ileo-colic presentation was significantly more marked in the southern states than in the western states (91.7% vs. 80.6%; *p* < 0.001). The intussusception was more commonly treated with radiological methods in the southern states (89.2% vs. 56.9%), whereas surgical and mixed methods were used significantly more frequently in the western states (43.1% vs. 10.8%; *p* < 0.001). The delay between onset and admission differed significantly between the two regions (*p* < 0.001), whereas the delay between onset and surgical treatment did not differ significantly by treatment modality (Table 1).

The majority of the children from both regions (78.4%) had received at least one dose of rotavirus vaccine, with no significant difference between the southern and western states (77.2% vs. 80.6%; *p* = 0.153). A higher proportion of children from the southern states were exclusively breastfed (99.4% vs. 95.5%; *p* = 0.045). The median [IQR] age at the time of initiation of solid foods was lower in the southern than in the western states (6 [5–6] mo versus 6 [5–8] mo; *p* < 0.001). There were four fatal cases, all of which occurred in the western states. Despite the death rate being less than 1% in the western states, the difference in the death rate by region was significant (0.9% vs. 0.0%; *p* = 0.022) (Table 1).

## Discussion

This analysis identified several differences in the epidemiology, clinical presentation, treatment, and outcome between children with intussusception in the southern (Kerala and Karnataka) and western (Maharashtra and Gujarat) states in India. Both these regions vary in their healthcare infrastructure and practices. The number of children enrolled was higher in the southern states than in the western states. Studies by Das et al. [3], and Bahl et al. [10] have also reported a high rate of intussusception in southern India. In this study, the median age of children with intussusception was slightly lower in the western states than in the southern states, consistent with the result of study by Mehendale et al. [15]. Additionally, in contrast with the findings of a study by Das et al. [3], the proportion of the affected children who were infants was higher in the western states than in the southern states.

The wealth distribution among the participants differed by region, with a higher proportion of children from the western states falling into the lower wealth tertile; Ghosh reported similar findings [11]. This raises the possibility that socioeconomic challenges may have created barriers in timely access to health care, contributing to delays in seeking treatment and the differences in outcomes. Similarly, more children from the southern states were treated in private hospitals, thus revealing disparities in wealth which in turn influence healthcare access, timing of intervention, and ultimately, the outcome.

The clinical presentation of intussusception in the southern and western states revealed some notable differences. Signs such as vomiting, abdominal tenderness, fever, and diarrhea, were more common in children from the western states than in those from the southern states, suggesting possible regional differences in the epidemiology of infections or underlying gastrointestinal conditions [14, 16]. Conversely, the incidence of incessant crying, a classic sign of intussusception, as a clinical presentation was notably more frequent in children from the southern states than in those from the western states [3, 17]. The predominant ileo-colic presentation of intussusception was observed in a markedly higher proportion of children from the southern states, consistent with the findings of a study by Das et al. [3].

Radiological methods, including hydrostatic, pneumatic, and liquid contrast, were more frequently used in the southern states than in the western states, whereas surgical methods, including manual reduction and resection, were used more frequently in the western states than in the southern states, consistent with reports by Reddy et al. [8]. These differences in management strategies may reflect regional differences in healthcare infrastructure, access to diagnostic tools, and treatment protocols.

The delay in seeking treatment was significantly longer in the western states, which supports the hypothesis that socioeconomic factors, such as distance to healthcare facilities, lack of awareness, and financial constraints, may delay the diagnosis and management of intussusception [18]. As intussusception is potentially life-threatening, early diagnosis and treatment are important to reduce the morbidity and mortality. Thus, measures are needed to increase awareness and improve healthcare access in rural and low-income areas.

A notably higher proportion of children from the southern states were exclusively breastfed, consistent with the higher rates of breastfeeding in the southern states reported by Valappil et al. [19]. The protective role of breastfeeding against gastrointestinal infections, which may contribute to intussusception, is well documented [20]. Additionally, the majority of children in both regions had received at least one dose of the rotavirus vaccine, which is likely to have reduced the risk of natural rotavirus infection and associated intussusception, as observed in a study by Reddy et al. [8].

Although the overall mortality was low (0.3%), the western region had four fatalities, whereas no fatalities were reported in the southern region, demonstrating a significant difference (*p* = 0.022). These findings suggest that, despite the regional differences in clinical presentation, diagnostic strategies, and socioeconomic factors, the outcome for children with intussusception is generally favorable in both regions [3]. Further research is warranted to explore potential contributing factors to regional differences in death rates, including delays in treatment, late presentation, and limited access to advanced care, as suggested by John et al. [17].

The multicenter design and large sample size using real-world data gives a detailed insight into the regional differences in the epidemiological profile, clinical characteristics and management strategies used. This thorough comparison suggests the impact of geographic, socioeconomic, and healthcare disparities on the outcome of intussusception. These results may help policymakers to design policies customized to each region.

However, this study has some limitations. All study sites in both regions were tertiary care hospitals; therefore, the results of this study do not reflect the incidence of intussusception in the community. There is a possibility of recall bias in the self-report of breastfeeding practices (especially in children older than 12 mo) and presenting complaints. Although the authors analyzed the methods of diagnosis and treatment, they did not investigate whether the cases were managed according to the guidelines or whether the study institutions had surgeons available at all times.

## Conclusions

This study revealed significant regional differences in the epidemiology, clinical presentation, management, and outcome of intussusception among children younger than 2 y in the southern and western states of India. These differences highlight the regional variations in healthcare infrastructure, complexity of managing pediatric intussusception, and socioeconomic disparities between the two regions. Managing these disparities through improved health education, better access to diagnostic tools, and timely intervention could reduce the burden of intussusception in different regions in India.

## Figures and Tables

**Table 1:** Baseline characteristics of children admitted with intussusception in the southern (Kerala and Karnataka) vs. western (Maharashtra and Gujarat) states in India

Characteristic	All (*N* = 1319) *n* (%)	Southern states (*N* = 860) *n* (%)	Western states (*N* = 459) *n* (%)	*p*
Age (mo), median (IQR)	9 (6–15)	9 (6–15.5)	8 (6–13)	**0.025** ^a^
Age group, *n* (%)				**0.001** ^b^
< 12 mo	840 (63.7)	520 (60.5)	320 (69.7)	
12–23 mo	479 (36.3)	340 (39.5)	139 (30.3)	
Sex, *n* (%)				0.474^b^
Male	845 (64.1)	545 (63.4)	300 (65.4)	
Female	474 (35.9)	315 (36.6)	159 (34.6)	
Hospital type, *n* (%)				0.170^b^
Government	771 (58.5)	491 (57.1)	280 (61.0)	
Private	548 (41.5)	369 (42.9)	179 (39.0)	
Wealth tertile, *n* (%)				**< 0.001** ^b^
Lower	498 (37.8)	280 (32.6)	218 (47.5)	
Middle	645 (48.9)	456 (53)	189 (41.2)	
Upper	176 (13.3)	124 (14.2)	52 (11.3)	
Admission to hospital, *n* (%)				**0.016** ^b^
Transfer from another hospital	471 (35.7)	327 (38.0)	144 (31.4)	
Direct admission	848 (64.3)	533 (62.0)	315 (68.6)	
Presenting complaint				
*Vomiting, n (%)* (*N* = *1309*)				**< 0.001** ^c^
Yes	998 (76.2)	616 (72.5)	382 (83.2)	
No	311 (23.8)	234 (27.5)	77 (16.8)	
*Abdominal tenderness*, *n (%)* (*N = 1285)*				**< 0.001** ^c^
Yes	874 (68.0)	534 (63.4)	340 (77.1)	
No	411 (32.0)	310 (36.6)	101 (22.9)	
*Blood in stools*, *n (%)* (*N= 1308)*				**< 0.001** ^c^
Yes	581 (44.4)	323 (38)	258 (56.3)	
No	727 (55.6)	527 (62)	200 (43.7)	
*Diarrhea*, *n (%)* (*N = 1306)*				**< 0.001** ^c^
Yes	295 (22.6)	138 (16.2)	157 (34.5)	
No	1011 (77.4)	713 (83.8)	298 (65.5)	
*Incessant crying and irritability*, *n (%)* (*N = 1319)*				**< 0.001** ^b^
Yes	283 (21.5)	277 (32.2)	6 (1.3)	
No	1036 (78.5)	583 (67.8)	453 (98.7)	
*Fever* (*N= 1300)*, *n (%)*				**< 0.001** ^c^
Yes	271 (20.8)	117 (13.8)	154 (33.8)	
No	1029 (79.2)	728 (86.2)	301 (66.2)	
*Constipation (N = 1304)*, *n (%)*				**< 0.001** ^c^
Yes	91 (7.0)	44 (5.2)	47 (10.4)	
No	1213 (93.0)	806 (94.8)	407 (89.6)	
Location of intussusception, *n* (%)				**< 0.001** ^c^
Ileo-colic	1159 (87.9)	789 (91.7)	370 (80.6)	
Colo-colic	53 (4)	14 (1.6)	39 (8.5)	
Ileo-ileal	41 (3.1)	13 (1.5)	28 (6.1)	
Multiple sites	17 (1.3)	6 (0.7)	11 (2.4)	
Unknown	49 (3.7)	38 (4.5)	11 (2.4)	
Management strategy, *n* (%)				**< 0.001** ^c^
Radiological reduction^d^	1028 (77.9)	767 (89.2)	261 (56.9)	
Surgical reduction	132 (10)	9 (1.1)	123 (26.8)	
Surgical resection	21 (1.6)	2 (0.2)	19 (4.1)	
Mixed modality^e^	138 (10.5)	82 (9.5)	56 (12.2)	
Post-treatment/surgical complications, *n* (%)				0.332^b^
Present	15 (1.1)	8 (0.9)	7 (1.5)	
Absent	1304 (98.9)	852 (99.1)	452 (98.5)	
Delay in seeking treatment (d), median (IQR)	1 (1–2)	1 (1–2)	1 (1–2)	**< 0.001** ^c^
Delay in seeking treatment (d) by treatment modality, median (IQR)				
Radiological reduction	1 (1–2)	1 (1–2)	1 (1–2)	0.080^a^
Surgical reduction	2 (1–3)	1 (1–2)	2 (2–4)	0.856^a^
Surgical resection	2 (2–3)	1.5 (1–2)	2 (1–3)	0.242^a^
Mixed	1 (1–2)	1 (1–2)	1 (1–2)	0.198^a^
Rotavirus vaccination status, *n* (%)				0.153^b^
Unvaccinated	285 (21.6)	196 (22.8)	89 (19.4)	
Vaccinated with ≥ 1 dose	1034 (78.4)	664 (77.2)	370 (80.6)	
Exclusive breastfeeding, *n* (%) (*N* = 311)				**0.045** ^b^
Present	304 (97.7)	178 (99.4)	126 (95.5)	
Absent	7 (2.3)	1 (0.6)	6 (4.5)	
Duration of exclusive breastfeeding (mo), median (IQR) (*N* = 304)	5 (4–6)	5 (4–6)	5 (4–7)	0.204^a^
Age at beginning solid food (mo), median (IQR) (*N* = 873)	6 (5–7)	6 (5–6)	6 (5–8)	**< 0.001** ^a^
Clinical outcome, *n* (%)				**0.022** ^c^
Survived	1307 (99.1)	853 (99.1)	454 (98.9)	
Transferred out	4 (0.3)	3 (0.4)	1 (0.2)	
Left against medical advice	4 (0.3)	4 (0.5)	0 (0.0)	
Died	4 (0.3)	0 (0.0)	4 (0.9)	

a Rank-sum test

b Chi-square test

c Fisher’s exact test

d Radiological reduction included hydrostatic, pneumatic, and liquid contrast medium;

e Mixed modality included any combination of radiological reduction, surgical reduction, and surgical resection
